# Brillouin–Raman microspectroscopy for the morpho-mechanical imaging of human lamellar bone

**DOI:** 10.1098/rsif.2021.0642

**Published:** 2022-02-02

**Authors:** M. Alunni Cardinali, A. Di Michele, M. Mattarelli, S. Caponi, M. Govoni, D. Dallari, S. Brogini, F. Masia, P. Borri, W. Langbein, F. Palombo, A. Morresi, D. Fioretto

**Affiliations:** ^1^ Department of Physics and Geology, University of Perugia, Via A. Pascoli, Perugia 06123, Italy; ^2^ Istituto Officina Dei Materiali, National Research Council (IOM-CNR), Unit of Perugia, c/o Department of Physics and Geology, University of Perugia, Via A. Pascoli, Perugia 06123, Italy; ^3^ Reconstructive Orthopaedic Surgery and Innovative Techniques – Musculoskeletal Tissue Bank, IRCCS Istituto Ortopedico Rizzoli, Via G.C. Pupilli 1, Bologna 40136, Italy; ^4^ Complex Structure of Surgical Sciences and Technologies, IRCCS Istituto Ortopedico Rizzoli, Via Di Barbiano 1/10, Bologna 40136, Italy; ^5^ School of Biosciences, Cardiff University, Museum Avenue, Cardiff CF10 3AX, UK; ^6^ School of Physics and Astronomy, Cardiff University, The Parade, Cardiff CF24 3AA, UK; ^7^ School of Physics and Astronomy, University of Exeter, Exeter EX4 4QL, UK; ^8^ Department of Chemistry, Biology and Biotechnology, University of Perugia, Via Elce di Sotto 8, Perugia 06123, Italy; ^9^ CEMIN - Center of Excellence for Innovative Nanostructured Material, Via Elce di Sotto 8, Perugia 06123, Italy

**Keywords:** Raman spectroscopy, Brillouin spectroscopy, bone imaging, biomaterial characterization, bone micromechanics

## Abstract

Bone has a sophisticated architecture characterized by a hierarchical organization, starting at the sub-micrometre level. Thus, the analysis of the mechanical and structural properties of bone at this scale is essential to understand the relationship between its physiology, physical properties and chemical composition. Here, we unveil the potential of Brillouin–Raman microspectroscopy (BRaMS), an emerging correlative optical approach that can simultaneously assess bone mechanics and chemistry with micrometric resolution. Correlative hyperspectral imaging, performed on a human diaphyseal ring, reveals a complex microarchitecture that is reflected in extremely rich and informative spectra. An innovative method for mechanical properties analysis is proposed, mapping the intermixing of soft and hard tissue areas and revealing the coexistence of regions involved in remodelling processes, nutrient transportation and structural support. The mineralized regions appear elastically inhomogeneous, resembling the pattern of the osteons' lamellae, while Raman and energy-dispersive X-ray images through scanning electron microscopy show an overall uniform distribution of the mineral content, suggesting that other structural factors are responsible for lamellar micromechanical heterogeneity. These results, besides giving an important insight into cortical bone tissue properties, highlight the potential of BRaMS to access the origin of anisotropic mechanical properties, which are almost ubiquitous in other biological tissues.

## 1. Introduction

Human lamellar bone is a highly specialized type of tissue, characterized by a complex hierarchical structure with several levels of organization, from the macroscopic scale, i.e. long, short and irregular bone; down to the mesoscale, i.e. cortical and trabecular bone; the microscale, i.e. lamellae, lacunae and the Haversian system; and the nanoscale, i.e. collagen type I and hydroxyapatite nanocrystals [1]. Cortical bone tissue is mostly located in the outer layer of the long bones and constitutes approximately 80% of the total skeletal mass. It is organized in regular and concentric patterns of lamellae, wrapped around a Haversian channel [2]. Recent works by Reznikov *et al*. have introduced the intriguing concept of a fractal-like structure, with increasingly smaller constituents retaining the characteristics of an ordered and disordered phase, that can be observed at each level of hierarchy [3–5].

A consequence of this sophisticated architecture is the dependence of the whole-structure performance on the synergy of all its constituents, starting from the nanoscale dimension. An impairment in just one single component at the nano-, sub-micro- or microscale can affect the biomechanics of the entire bone material [6,7]. For this reason, considering the lamellar organization at each length scale is fundamental both for understanding its complex mechanical properties and for modelling prostheses and bioinspired materials [8,9]. In addition, knowledge of bone tissue physiology can be crucial to detecting micro-alterations in the structure that can be early manifestations of orthopaedic diseases [10].

Several techniques with different spatial resolutions have been employed to assess bone mechanics. However, since bone is both inhomogeneous and anisotropic, the mechanics at the micrometric and sub-micrometric scales is still not thoroughly understood, leaving open the discussion about the origin of micromechanical modulation in bones. In fact, ultrasound-based techniques [11–13], such as scanning acoustic microscopy, and nano-indentation [14,15] performed on cortical bone have revealed a certain degree of micromechanical modulation, but its origin is still controversial. Because of this, several questions remain open, especially concerning the role of packing and orientation of the mineralized bundles and the role of the degree of mineralization of the collagen bundles in the local modulation of stiffness [16]. In addition, traditional micromechanical investigations are blind to the local chemical composition of bones, so that the molecular origin of mechanical modulation has not yet been investigated in depth.

In the present work, we address these fundamental issues by analysing a highly mineralized type of lamellar bone, namely cortical bone, by an innovative technique, Brillouin–Raman microspectroscopy (BRaMS), which is contactless, non-destructive and label-free, and enables the micromechanical and chemical properties of materials to be assessed without altering or damaging their structure [17,18]. The proposed method merges two microspectroscopy techniques in the same instrumental set-up: Brillouin scattering [19–23], which is an emerging tool in the biomechanics field, and Raman scattering [24,25], which is a well-established vibrational approach for the chemical characterization of biological samples. The combination of these techniques gives us the unique capability to investigate the relation between the mechanical properties of a biological system and the underlying molecular structure and composition. In recent years, BRaMS has been successfully used to investigate structured materials [18], collagen hydrogels [26], single-cell mechanics [27–29], microbial biofilms [30,31] and various biological tissues [22], including amyloid plaques in the brain in Alzheimer's disease [32] and human cornea [33]. Moreover, we previously investigated the chemo-mechanical properties of the head and diaphysis of the human femur, demonstrating the possibility of analysing both frozen and formalin-fixed tissue sections with high reproducibility [34,35].

Here, we report a wide-area (0.25 × 0.4 mm) BRaMS map of cortical bone, which enables the simultaneous investigation of the lamellar architecture contributions to the mechanical and chemical properties of the whole osteon's structure. Our results, aided by innovative hyperspectral data analysis tools, reveal a complex biomechanical landscape, much richer than previously thought. In particular, the Brillouin spectra of the Haversian systems present different contributions related both to the disordered phase of the extracellular matrix (ECM) and to the ordered phase of collagen bundles with different degrees of mineralization. The relative intensity of Brillouin peaks gives a label-free histological map of the tissue, revealing the morphology of the main constituents of the osteonal systems, while the map of Brillouin frequency shifts shows the stiffness distribution, characterized by micrometric gradients induced by the lamellar structure of mineralized bundles. Moreover, Raman imaging as well as scanning electron microscopy (SEM) with energy-dispersive X-ray (EDX) analysis help to clarify the role of mineralization in the establishment of the characteristic lamellar pattern.

## 2. Results and discussion

### 2.1. Local mechanical and chemical heterogeneities revealed by single Brillouin–Raman microspectroscopy spectra

**Figure 1.**
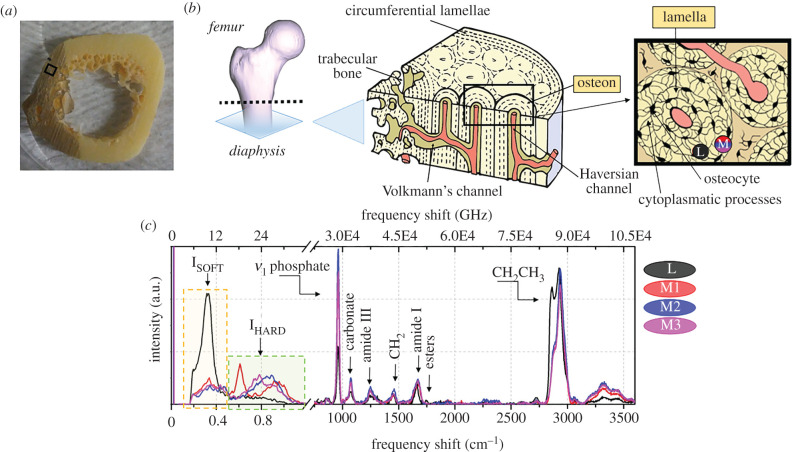
(*a*) Cross-section of a femur diaphysis with (*b*) a schematic description of its histological features. Black box: osteons or Haversian systems in the cortical bone region where all the measurements were collected. (*c*) Typical Brillouin and Raman spectra were collected in a lacuna (L, black) containing the cells and in different points (M1, red; M2, blue; M3, magenta) of the ECM forming the lamellar structure.

BRaMS measurements were performed on the cross-section of a human diaphysis, as shown in figure 1*a*. The typical structural features of the cross-sectional diaphysis include a variety of tissue organizations ranging from the external periosteum to the internal trabecular region (figure 1*b*). In this study, we focused our attention on a region of the cortical bone which is characterized by the so-called Haversian systems or osteons. Each osteon is formed by a series of concentric lamellae, composed of a mineralized ECM wrapped around a central blood vessel called the Haversian channel. The cells, i.e. osteocytes, remain confined into non-mineralized ellipsoidal spaces, called lacunae, and communicate with one another through cytoplasmic protrusions enveloped inside a mesh of canaliculi. The boundary of each osteon is defined by optically dense structures, called cement lines, and the space between different Haversian systems is filled with interstitial lamellae, i.e. the residues of old osteons partially degraded by bone remodelling processes.

Figure 1*c* shows the inelastic scattering spectra, with both the Brillouin (before the break) and Raman (after the break) characteristic signatures of cortical bone tissue. Spectra were collected from the lacunar space (L, black) and the extracellular mineralized matrix forming the lamellar structure (M1, red; M2, blue; M3, magenta). Raman peaks originate from the vibrational modes of the molecular chemical bonds interacting with the laser beam, thus providing a chemical fingerprint of bone tissue, whereas each peak in the Brillouin spectrum gives the characteristic frequency $\nu_{B}$ of an acoustic mode propagating through a fraction of the micrometric scattering volume of the sample illuminated by the confocal microscope (see Material and methods). The hardness of matter supporting the acoustic mode is expressed by the longitudinal elastic modulus, *M*, which is proportional to the square of the Brillouin frequency shift, $\nu_{B},$through the relation

2.1 $$M=\frac{\nu_{B}^{2}\;\;\lambda^{2}\rho }{4n^{2}},$$

where *ρ* is the mass density, *n* is the refractive index of the medium and *λ* is the wavelength of the laser source. It is worth noting that, since it is possible to detect different peaks in a single spectrum, the micro-heterogeneity revealed by distinct peaks has a granularity that is larger than several hundred nanometres, which is just sufficient to support the propagation of the acoustic mode [36,37].

In our previous work [34,35] using the same excitation wavelength and scattering geometry, we isolated two main regions in the bone Brillouin spectrum: the first one was attributed to a ‘soft’ phase, ranging between 4 and 13 GHz (I_SOFT_; yellow box in figure 1*c*), and the second to a ‘hard’ phase with peaks ranging between 17 and 34 GHz (I_HARD_; green box). These two spectroscopic features were attributed to the coexistence within the probed scattering volume of a soft disordered phase of ECM, non-collageneous proteins and poorly oriented fibres, which are the main components of the lacunae–canaliculi system, and a hard ordered phase of collagen bundles with various degrees of mineralization. The average spectral parameters of intensity and frequency shift were calculated through the zero and first spectral moments for both soft and hard components [35].

In the present work, which focuses on cortical bone, we take an essential step forwards in the interpretation of Brillouin spectra, guided by the evidence of the coexistence of distinct contributions to the hard phase. In fact, whereas the typical Brillouin spectrum collected in the lacunar niche shows a clear peak at about 8 GHz, ascribed to the non-ordered or soft phase of bone tissue P_SOFT_ (yellow box of figure 1*c*), the Brillouin spectra collected in different regions of the ECM are characterized by an evident broadening of the hard phase spectral shape, due to a heterogeneous contribution to the Brillouin linewidth. Among these peaks, the one centred at about 18.5 GHz can be easily separated from the other contributions in some spectra (figure 1, M1). This component resembles the features of the non-calcified layer of subchondral bone [34], where Raman spectra show the typical signature of non-mineralized collagen fibres. It is worth noting that the coexistence of the 18.5 GHz and the mineralized matrix peak in the same Brillouin spectrum suggests that some regions of the osteon's ECM are composed of both mineralized and non-mineralized collagen bundles organized in aggregates of sub-micrometric size [37]. Lastly, a small contribution centred at about 14 GHz can be detected, whose origin has yet to be clarified.

This rich and complex spectral scenario led us to develop a data analysis method specifically designed to decompose each Brillouin spectrum into multiple peaks, assigning each peak to a distinct local (micrometric average) elastic environment, as described in the Material and methods. Specifically, the multiple-peaks fitting procedure consists of a nonlinear least-squares fitting of the sum of four distinct damped harmonic oscillator (DHO) peaks, namely P_SOFT_, P_14 GHz_, P_18 GHz_ and P_MINERALIZED COLL_, designed to take into account the existence of the above-mentioned four different spectral features. In addition to that, a hyperspectral analysis based on a recently introduced unsupervised non-negative matrix factorization (NMF) method [38] is also explored. This method, using information contained in thousands of spectra, reveals additional signatures that are too weak to be extracted from a single spectrum (see Material and methods), thus providing a further, complementary, approach to the multiple-peak fitting.

### 2.2. Brillouin–Raman microspectroscopy chemo-mechanical mapping reveals the structure of the Haversian systems

**Figure 2.**
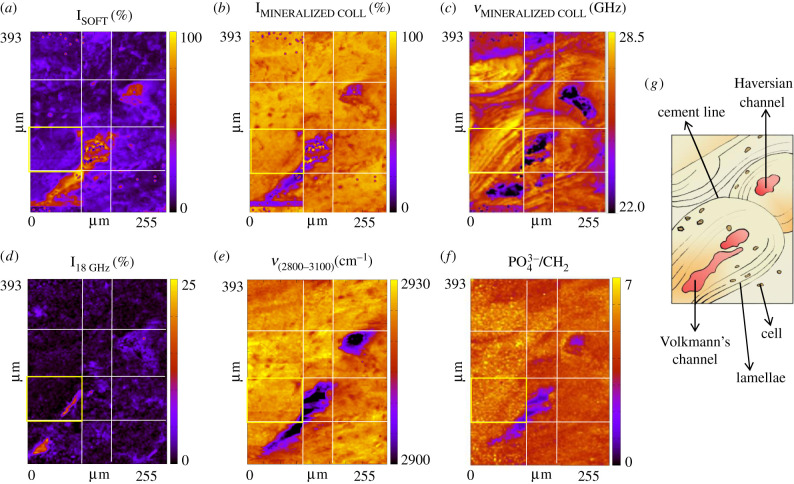
BRaMS imaging of cortical bone reveals the architecture of the Haversian systems, by the maps of the spatial distribution of (*a*) the relative volume fraction of the soft component (I_SOFT_); (*b*) the relative volume fraction of the mineralized collagen bundles (I_MINERALIZED COLL_) and their (*c*) frequency shift (*ν*_MINERALIZED COLL_); (*d*) the relative volume fraction of collagen bundles (I_18 GHz_); (*e*) the first spectral moment of the Raman CH stretching peak (*ν*_(2800–3100)_) estimating the variation of the lipid-to-protein ratio; (*f*) the ratio of Raman ${\mathrm{PO}}_{4}^{3-}$ at 959 cm^−1^ to CH_2_ wagging at 1465 cm^−1^ (${\mathrm{PO}}_{4}^{3-}/CH_{2}$ ) estimating the mineral-to-matrix ratio, i.e. the bone mineralization degree, and (*g*) a schematic illustration of the main biological structures recognizable in the map. White lines denote tiles that compose the mosaic images.

To further our understanding of the Brillouin and Raman spectral shapes detected in single-point spectra, the fine microstructure of cortical bone was investigated using BRaMS mapping as described in the Material and methods. Brillouin and Raman hyperspectral maps obtained from the cortical bone are reported in figure 2, revealing the spatial distribution of the main characteristic biological units constituting the Haversian system, i.e. cells and lacunae, mineralized collagen bundles and regions of bone remodelling. BRaMS maps highlight all these features using an endogenous contrast mechanism, i.e. the elastic properties and the concentration of molecular groups revealed by Brillouin and Raman spectra, respectively. The intensities of the Brillouin peaks P_SOFT_, P_14 GHz_, P_18 GHz_ and P_MINERALIZED COLL_, obtained following the multiple-fit procedure described in the Material and methods, were normalized both considering for each spectrum the coexistence in the same scattering volume of soft and hard phases within the scattering volume and weighting their scattering efficiency by following the procedure explained in [35]. The results, namely I_SOFT_, I_14 GHz_, I_18 GHz_ and I_MINERALIZED COLL_, describing the volume fraction occupied by each contribution to the micro-heterogeneity, are reported in figure 2, together with the frequency shift of the peak related to the mineralized collagen bundles (*ν*_MINERALIZED COLL_), also obtained by the fitting procedure. Moreover, the first spectral moment of the CH_2_CH_3_ stretching (*ν* [2800–3100]) Raman band is also reported in figure 2, providing the distribution of the lipid-to-protein ratio [27,35], together with the ratio of the Raman intensity of the ${\mathrm{PO}}_{4}^{3-}$ peak at 959 cm^−1^ to that of the CH_2_ wagging peak at 1465 cm^−1^ (${\mathrm{PO}}_{4}^{3-}/CH_{2}$), giving a measure of the mineral-to-matrix ratio, i.e the degree of bone mineralization [39].

Figure 2 noticeably shows that maps based on the intensity of Brillouin peaks represent all the typical features of the cortical bone, providing a mechanical depiction of the Haversian systems. Specifically, two adjacent osteons linked by a Volkmann channel in the bottom left part of the maps are apparent in this region, together with an osteon wrapped around a Haversian channel in the top right part. In particular, figure 2*a* displays the intensity map of the matrix component (I_SOFT_), which is mainly concentrated inside the lacunae and the Haversian and Volkmann channels. Some interspersed matrix around the vessels and the lacunae can be attributed to the presence of the mesh of canaliculi, which are the structural niches formed by the disordered phase that are responsible for the cells' survival and metabolic exchanges. Conversely, figure 2*b* shows the map of the hard component (I_MINERALIZED COLL_), i.e. the ECM composed by the ordered phase of mineralized collagen bundles, the main constituent of cortical bone. A strong similarity is found between the features visible in these two maps and those revealed by the distribution of the first spectral moment of the Raman CH stretching band at 2800–3100 cm^−1^ in figure 2*e*. In fact, in the latter map, a shift to lower frequency values corresponds to an increase in the lipid-to-protein ratio, given that the lipid CH_2_ stretching is centred around 2880 cm^−1^ while the protein CH_3_ stretching lies around 2935 cm^−1^ [31]. This suggests that in the disordered phase of lacunae and canaliculi, as well as in the centre of the channels, lipids from the cell membranes are present, contributing to the soft component (I_SOFT_), while the collagen bundles forming the ECM and thus contributing to the hard component (I_MINERALIZED COLLAGEN_) are the main protein constituents of cortical bone.

Figure 2*d* reports the map of the I_18 GHz_ component distribution, disclosing that it is visible uniquely within some very localized regions inside the mineralized ECM, specifically around the central blood vessels in both Volkmann and Haversian channels, and close to some lacunar–canalicular nodes. Since the correlated Raman signature is typical of the non-mineralized collagen (data not shown), this elastic component is likely to have originated from sites of bone remodelling, according to the idea that the first ECM deposited by the osteoblasts contains fibres of non-mineralized collagen, which only later undergo a mineralization process via enzymatic reactions highly regulated by signals originating from osteocytes [40–42]. Therefore, this 18 GHz Brillouin signal may be considered a key parameter for bone tissue characterization, since ECM remodelling is involved in several physiological processes (e.g. fracture repair and prosthetic integration after a replacement), as well as in orthopaedic diseases related to bone fragility (e.g. osteoporosis, osteopenia). Note that the capability of Brillouin spectroscopy to discriminate between the mechanical characteristics of different sub-micrometric components, such as bundles of mineralized and non-mineralized collagen, is a great advantage over ultrasonic techniques, which at the microscale can only provide an estimate of the average properties of the ECM.

Finally, a Raman map of the mineral-to-matrix ratio was obtained by plotting the ratio between phosphate stretching ($\nu_{1}{\mathrm{PO}}_{4}^{3-}$) and CH_2_ wagging integrated intensities in figure 2*f*. The latter peak was chosen to represent the organic component of bone since it is stable in shape in different regions of the sample, and it provides a better signal-to-noise ratio than other signatures of collagen, such as the proline and hydroxyproline vibrations at 855 and 876 cm^−1^. The mineralization degree distribution is visibly related to the I_MINERALIZED COLL_ map, i.e to the presence of the ordered bundles; however, it does not reproduce the typical lamellar pattern of osteons. Instead, figure 2*c,* showing the frequency shift of the mineralized collagen bundles (*ν*_MINERALIZED COLLAGEN_), clearly draws the typical concentric shape of lamellae wrapped around the vessels. To our knowledge, this is the first application of Brillouin spectroscopy for the morpho-mechanical depiction of the lamellar pattern in human cortical bone, revealing the periodic modulation in the osteon's elastic properties. The graphical sketch in figure 2*g* summarizes the main biological structures recognizable on the map.

### 2.3. Brillouin frequency shift of mineralized bundles and morpho-mechanics of osteon lamellar patterns

**Figure 3.**
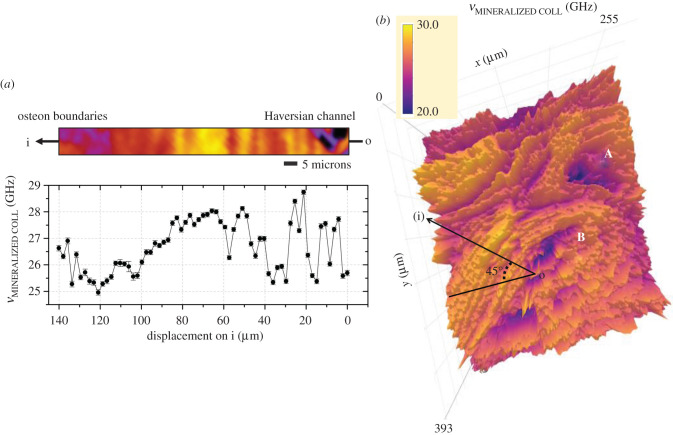
(*a*) Morpho-mechanical characterization of the osteon lamellar pattern, showing the modulation in Brillouin frequencies through the diagonal region (i) delimited in (*b*); (*b*) map of the distribution of the Brillouin frequency shift (*ν*_MINERALIZED COLL_).

Figure 3 reports the map of the frequency shift of the Brillouin peak attributed to the phonons propagating through mineralized collagen (*ν*_MINERALIZED COLL_) to investigate more deeply the morpho-mechanical characteristics of the lamellar pattern. First, we notice that the lamellar morphology is very different between Haversian systems, confirming the notion that, also in very localized regions of the cortical bone, the osteons are heterogeneous in both size and shape. In particular, the lamellae in osteon B are wider and more pronounced than those in osteon A. A portion of this picture, perpendicular to the lamellae of osteon B (i), was selected from the map and reported in figure 3*a* to evaluate the average lamellar thickness, revealing a distribution between *ca* 4 and 14 µm, with an average value of 7 µm, which is in the expected range for these bones [43]. In this region, the Brillouin frequency shift (lower part of figure 3*a*) shows a variation of about 15%, ranging between 24.5 and 28.5 GHz, well beyond the data uncertainty. This corresponds to a range of variation of about 30% for the longitudinal elastic modulus *M*, namely from 35.4 to 47.8 GPa, obtained taking *ρ* = 2 g cm^−3^ and *n* = 1.55 and assuming a constant ratio *ρ*/*n*^2^ through the sample, as in [35]. In this regard, it is interesting to notice that a variation of about 30% was also observed for the longitudinal elastic modulus of dry collagen for parallel (18.6 GPa) and orthogonal (14.3 GPa) directions to the fibre axis [44]. We thus ascribe the modulations observed in figure 3 to different orientations of mineralized collagen bundles. It is worth noting, however, that considering the ratio *ρ*/*n*^2^ to be constant in the calculation of the elastic modulus, while proven to be reasonable in collagen gels at various concentrations [26], may not be a good approximation in some regions of the sample where the chemical composition becomes much more heterogeneous (e.g. in Haversian channels) or the direction of the fibres abruptly changes, thus influencing the value of the longitudinal elastic modulus. Because of this, since Raman spectroscopy can be a potentially powerful tool for uncovering and accounting for local variations in *ρ*/*n*^2^ [45], a more detailed discussion of the longitudinal elastic modulus variations in cortical tissue will be the subject of future works.

Moreover, a lower frequency shift is found close to Haversian canals and between adjacent osteons, i.e. the cement lines. This is in accordance with the results obtained by Burr *et al*., who found in the cement lines a relatively ductile interface between the osteon and its surroundings [46], and it might be attributed to the presence in these regions of a more disordered mineralized phase.

A similar mechanical heterogeneity in the cortical bone has also been observed by other micro-mechano-sensitive techniques, such as nano-indentation and scanning acoustic microscopy, but the explanation about the origin of this modulation is still unclear. In fact, in some works, it has been ascribed to the presence of arrays of mineralized bundles with different orientations [16,47], while, in other works, it has been attributed to alternative lamellae with different degrees of mineralization or to an alternate degree of packing of mineralized fibrils in the bundles (the so-called dense–loose motif) [48–51]. The contrast between these different interpretations is the cause of an objective difficulty in elaborating an exhaustive model of the osteon's lamellar microstructure. In this respect, our results support the idea that the mechanical changes found among adjacent lamellae are due to the presence of bundles with different orientations. In fact, in the other two possible cases of different degrees of mineralization or different packing fractions, the simultaneous Raman map of the phosphate-to-matrix ratio in figure 2*f* should have shown a patterned structure analogous to that of figure 2*c*, which is not the case.

**Figure 4.**
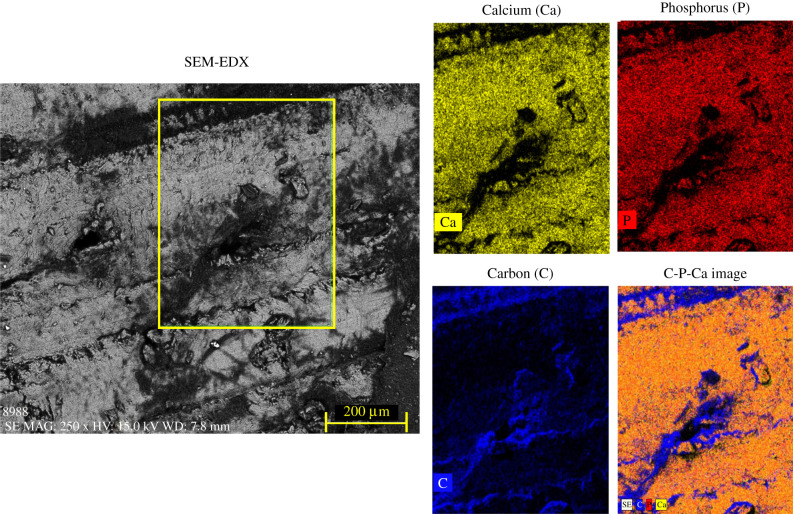
(*a*) Secondary electrons image of the cortical bone and the region observed with EDX enclosed in the yellow box. (*a*) Elemental mapping of (*b*) calcium (yellow, Ca), (*c*) phosphorus (red, P) and (*d*) carbon (blue, C), and (*e*) the overlaid image of the EDX analysis performed on the rectangular region corresponding to the zone mapped previously with BRaMS.

To validate our interpretation, the bone chemical composition was measured independently by EDX analysis, employing SEM (see Material and methods). The results shown in figure 4 illustrate the mapping of the most abundant elements retrieved in the X-ray spectral analysis, namely calcium (yellow), phosphorus (red) and carbon (blue), together with the overlaid EDX map and the region of interest enclosed in the yellow box of the secondary electrons image. The elemental analysis shows an almost homogeneous and interspersed distribution of the calcium and phosphorus content, with no apparent sign of a lamellar pattern, which would result from varying concentrations of the hydroxyapatite crystals, in line with the previous findings obtained from Raman analysis. We can therefore conclude that the mechanical modulation found by Brillouin spectroscopy can be mainly attributed to alternating arrays of mineralized bundles with different orientations.

### 2.4. The 14 GHz Brillouin mode and its correlation with mineralized lamellae explored by non-negative matrix factorization analysis

**Figure 5.**
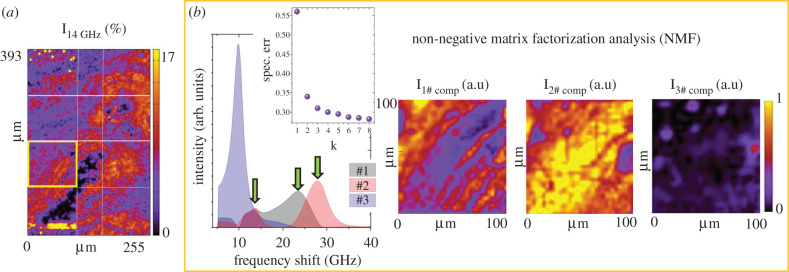
(*a*) Spatial distribution of the volume fraction of the I_14 GHz_ component; (*b*) NMF applied to the Brillouin map enclosed in the yellow box describes the dataset as a combination of three components, namely 1# component (black), 2# component (red) and 3# component (blue), whose spatial maps are reported. The green arrows point to the I_14 GHz_ component and the I_MINERALIZED COLLAGEN_, which are co-localized in components 1 and 2. The inset reports the reduction of the factorization spectral error with respect to the increasing number of components used in the factorization procedure.

The distribution of the P_14 GHz_ mode of the Brillouin spectrum is reported as volume fraction I_14 GHz_ in figure 5*a*, showing that this signal can be detected in the mineralized ECM of both osteons and suggesting its attribution to the mineralized collagen bundles (I_MINERALIZED COLL_).

To further the interpretation of the complex multi-peak structure of the bone Brillouin spectra, and to elaborate on the origin of the P_14 GHz_ mode and its possible correlation with longitudinal modes propagating within the mineralized lamellae, we have also modelled the whole set of Brillouin data forming the map, as a linear combination of a few components using the non-NMF approach [38] described in the Material and methods. This method, which has already proved capable of decomposing Brillouin maps from Alzheimer's brain tissue, while assigning less statistical significance to peaks sporadically expressed in the dataset, has the merit of extracting information across an entire set of spatially resolved spectra, such that otherwise faint or superimposed contributions can be disentangled. Furthermore, NMF performs a correlative treatment of different spectral contributions, allowing different Brillouin peaks to be attributed to the same elastic species within the sample. This is typically done by multivariate analysis (e.g. principal components analysis) in molecular spectroscopy but very rarely attempted in Brillouin light scattering. Figure 5*b* shows the three components determined by analysing the region inside the yellow box in figure 5*a*, numbered with decreasing average concentrations in the dataset and the corresponding concentration maps. We found that three components can suitably describe our spectra and that adding further components does not improve significantly the spectral error, as shown in figure 5*b,* inset.

Note that the I_18 GHz_ peak of the non-mineralized collagen was not singled out by NMF, confirming that its presence in the tissue is very scarce. Moreover, we can see that components 1 and 2 reveal the distribution of mineralized collagen bundles in the ECM, while component 3, which describes the distribution of the soft constituent, is mainly localized within lacunae and canaliculi. More specifically, component 1 shows a maximum at about 24 GHz and a long tail towards lower frequencies. According to our previous multi-peak analysis, it can thus be associated with both tilted bundles within osteonal lamellae and the hard phase close to canals and to the interstitial regions between adjacent osteons. On the other hand, component 2 is centred at about 27 GHz, describing the behaviour of the bundles that are almost orthogonal to the sample surface (i.e. parallel to the phonon wavevector). We notice that this decomposition method expresses the hyperspectral data as a linear combination of components defined by their spectral and spatial distribution. We can thus deduce that a gradual modulation of frequency passing from one lamella to another is here reconstructed as modulation in the intensity of the first and second components. This modulation is visible in the first two maps of figure 5*b*. Additionally, we notice that both components 1 and 2 show a small peak centred around 14 GHz, providing further indication of the co-localization of the 14 GHz spectral feature with the P_MINERALIZED COLL_ component generated by light scattered from longitudinal acoustic modes propagating through mineralized collagen. As a consequence, if the mineralized collagen elastic medium has two acoustic modes simultaneously visible via Brillouin scattering, the low-frequency one should be a transverse mode. The associated shear elastic modulus can be deduced by

2.2 $$G=\nu_{BT}^{2}\frac{\lambda^{2}\rho }{4n^{2}}\;\;,$$

with *v*_*BT*_ = 14 GHz, giving *G* = 11.5 GPa. This finding is also consistent with previous quasi-static (indentation) characterizations of bones, in which the ratio between longitudinal and transverse elastic moduli was also found to range between 3 and 4. The absolute values of the moduli were about 30% lower than those reported in the present work, possibly because of acoustic dispersion [52]. It is also interesting to notice that, though it is quite unusual to have Brillouin peaks in backscattering geometry from transverse acoustic modes, this is indeed reasonable for our sample where lamellar structures have anisotropic elastic and photoelastic matrices, thus supporting light scattering from transverse modes. In fact, at least two scattering mechanisms can be easily recognized in fibrous systems, typically associated with hexagonal crystalline symmetries [53,54]: (i) depolarized scattering from pure transverse modes, with polarization orthogonal to the extraordinary axis, due to off-diagonal photoelastic constants in the low-symmetry hexagonal systems, and (ii) polarized scattering from quasi-transverse acoustic modes, generating density fluctuations similar, even if less intense, to those associated with quasi-longitudinal acoustic modes.

## 3. Conclusion

In the present work, we investigated the potential of BRaMS by mapping a relatively wide area (0.25 × 0.4 mm) of human cortical bone including Haversian systems, a challenging workbench to test the potential of this correlative technique for the micromechanical and chemical characterization of specialized tissues. In fact, bone tissue is very complex at the micrometric and sub-micrometric level, enabling an unusually rich pattern of spectroscopic features in the Brillouin spectrum, which has been analysed by two complementary data analysis methods: a multi-peak least-squares fitting procedure and an unsupervised non-NMF method. BRaMS hyperspectral images have revealed the distribution and shape of all the relevant bone tissue components at the microscale, such as the lacunar–canalicular mesh, the blood vessels and the mineralized matrix distribution in the osteonal lamellar pattern, and have provided the chemo-mechanical properties of all these features in a contactless and non-destructive way. Moreover, BRaMS maps were able to discriminate between mineralized and non-mineralized collagen bundles, allowing us to monitor the regions more involved in bone remodelling processes and paving the way for the study of bone fracture repair mechanisms or prosthetic integration, as well as orthopaedic diseases that involve the matrix constituents' rearrangement. An extremely complex scenario in the mechanical properties of the mineralized matrix was revealed—a scenario that is induced by the anisotropy of the micrometric lamellar pattern of cortical bone. The combined Brillouin and Raman analyses, supported by EDX elemental analysis, show that the modulation of the elastic modulus in the lamellar pattern is not due to a different degree of mineralization, as proposed in previous mechanical investigations [50,51], but rather to the oscillating orientation of bundles when passing through adjacent lamellae, thus supporting the notion previously suggested by scanning acoustic microscopy [16]. Finally, the appearance of transverse acoustic modes in the Brillouin spectra, which can be ascribed to the anisotropy of the mineralized bundles, can considerably improve the elastic characterization of the tissue.

In conclusion, BRaMS revealed its potential in bone tissue characterization, providing a thorough evaluation of its morphology, chemical composition and mechanical behaviour at the microscale in a non-destructive and label-free manner. In particular, the morpho-mechanical and molecular investigations reported here are the first, necessary step in the application of BRaMS for histopathological analysis and the clinical translation of this technique for bone disease detection and monitoring.

### 3.1. Material and methods

#### 3.1.1. Sample preparation

The measurements were conducted on a cross-section of 5 mm thickness obtained from the right human femoral diaphysis, approximately 2 cm below the lesser trochanter, as shown in figure 1*a*. Specifically, the sample was collected from a cadaveric donor (male, 44 years of age) without a history of bone disorders by the Musculoskeletal Tissue Bank of IRCCS Istituto Ortopedico Rizzoli (Bologna, Italy; EU TE Code: IT000096) as previously reported in [35]. In this study, only a specimen not suitable for transplantation and considered as waste material was used, according to the Italian Legislation and National Transplantation Center's guidelines. Then, the sample was fixed in 4% buffered paraformaldehyde (PFA; Sigma-Aldrich) for 24 h, washed in running tap water and distilled water and stored at room temperature in a 70% alcohol/water mixture which does not damage the sample owing to prior PFA fixation. Before measurement, the sample was removed from the alcohol/water mixture and air-dried at room temperature.

#### 3.1.2. Brillouin–Raman microspectroscopy

The BRaMS set-up consists of a 532 nm single-mode solid-state laser, a polarizing beamsplitter, which reflects the laser light into a 20× (NA 0.42) microscope objective lens for imaging samples. The chosen optical configuration has a lateral spatial resolution of about 2 µm, while the axial resolution measured by the Brillouin Edge Spread Function on the bone tissue is about 15 µm. More details of the instrumentation can be found in [17,35]. Then, the backscattered light is split in frequency and direction by an edge filter, so that the Stokes component (greater than 30 cm^−1^) is sent to a Horiba iHR320 Triax Raman monochromator (Kyoto, Japan) and the quasi-elastic and anti-Stokes components (less than 30 cm^−1^) are sent to a high-contrast multi-pass tandem Fabry–Perot interferometer (TFP-2 HC; JRS Scientific Instruments, Zürich, Switzerland). The sample is mounted on an *xyz* translation stage for mapping. Twelve spatially sequential maps were performed in the cortical region of the diaphysis cross-section, by raster scanning with a 3 µm step size. Then, the partial maps were stitched together, resulting in the whole image of the lamellar systems covering a surface of about 393 × 255 µm^2^. Each Brillouin–Raman spectrum was collected for about 55 s, using a 600 lines/mm grating and 100 µm slit aperture (Raman resolution: ~10 cm^−1^, Brillouin resolution: ~100 MHz). Laser power on the sample was lower than 7 mW to prevent photodamage.

#### 3.1.3. Multiple-peaks Brillouin data analysis

**Figure 6.**
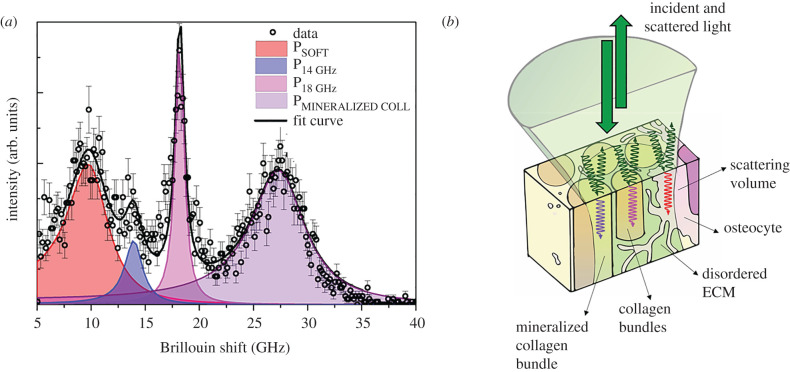
(*a*) Typical cortical bone Brillouin spectrum with the cumulative fitting function and its four distinct DHO components. (*b*) Schematic description of a micrometric region of the sample illuminated by the incident light, and Brillouin scattering processes arising from different constituents of the bone tissue.

The typical Brillouin spectrum collected on bone tissue and shown in Figure 1*b* (before the break) is composed of peaks ranging between 4 and 34 GHz. In our previous work, we isolated two main regions in the Brillouin spectrum: the first one attributed to a ‘soft’ phase, ranging between 4 and 13 GHz (I_SOFT_; yellow box in figure 1*b*), and the second to a ‘hard’ phase with peaks ranging between 17 and 34 GHz (I_HARD_; green box). The average spectral parameters of intensity and frequency shift were calculated through the zero and first spectral moments for both soft and hard components [34,35]. In the present work, we aim to reveal the fine structure of these complex spectral structures, decomposing them into single peaks and identifying their microscopic origin, as highlighted in figure 6*a*.

The intensities and frequency shifts obtained are used to map the elastic properties of the bone components. In this scheme, every single Brillouin peak is fitted to a DHO function [29]:

3.1 $$I(\omega )=\frac{I_{0}}{\pi }\;\frac{\omega_{B}^{2}\Gamma }{{\left(\omega^{2}-\omega_{B}^{2}\right)}^{2}+\omega^{2}\Gamma^{2}}.$$

The frequency shift of the Brillouin peak is $v_{B}=\omega_{B}/2\pi$, the linewidth is Γ/2π and the area is $I_{0}$. A preliminary fit was performed by using just the sum of two DHO peaks, one for the soft component centred at about 8 GHz and the other one for the hard component centred at about 24 GHz. This preliminary analysis showed large residuals in some of the spectra owing to the presence of at least two additional components at intermediate frequencies, namely around 14 and 18.5 GHz (which are also visible in the raw data of figure 1*b*). Based on several single fits of these two components, the substantial invariance of their parameters (peak position and width) was observed. The fitting algorithm of the whole spectrum was therefore based around four independent DHO peaks, each of them convoluted with the instrumental response: peak P_SOFT_ describing the soft component at around 8 GHz, peak P_14 GHz_ at a fixed frequency (*v*_B_ = 14 GHz) and width ((Γ/2π=2 GHz), peak P_18 GHz_ at a fixed frequency (*v*_B_ = 18.5 GHz) and width (Γ/2π=1 GHz), and peak P_MINERALIZED COLL_ describing the hard component at around 24 GHz. As the peak intensities of the four peaks were free fitting parameters and the background was negligible, the total number of varying parameters was eight. Considering the well-separated experimental peaks, a robust fitting procedure based on the quasi-Newton Levemberg–Marquardt method was automated for the analysis of the 102 × 136 spectra constituting the whole map.

*Non-NMF:* Brillouin maps are also explored using the unsupervised non-NMF method [38,55]. In the NMF approach, the data D are reshaped as a matrix of *s* × *p* dimensions, where *s* indicates the number of spectral points and *p* is the total number of spatial pixels in the image. The factorization decomposes the matrix into two non-negative matrices S, of dimensions *s* × *k*, and C, of dimensions *k* × *p*, where *k* indicates the number of components. The S matrix columns describe the spectra of the components found in the image, with spatially resolved concentration maps given by the corresponding C matrix rows. Starting from random seeds, i.e. without any prior knowledge of the nature of the components, the algorithm iteratively varies S and C to minimize the factorization error (defined as the Frobenius norm of D-S × C). Here, the procedure was repeated several times using eight random initial guesses and producing the same results, thus demonstrating high reproducibility. The number of components used in the factorization is increased until the error does not decrease significantly, with the retrieved components displaying unique spatial and spectral features (see the inset of figure 5*b*). In our analysis of the Brillouin data, we found that three components are sufficient to describe the hyperspectral images.

#### 3.1.4. Raman data analysis

Raman spectroscopy probes light inelastically scattered by the vibrational modes of the chemical species present within the sample. Owing to its high molecular specificity, Raman spectroscopy is suitable for analysing the chemical composition of biomaterials. Peak intensities are proportional to the concentration of molecular species so that probing the modulation of peak intensities in different regions of a sample provides maps with contrast based on molecular composition. The analysis of Raman spectra was performed using in-house software. Before the analysis, a pre-processing step was performed for which a background, fitted to a spline accounting for dark counts and luminescence, was subtracted from each acquisition. Then, the integrated intensity of some selected Raman peaks was calculated. Since the intensity of the spectrum can be influenced by different phenomena, such as fluctuations of the laser intensity in time or uncertainty in the scattering volume owing to the rough surface of the sample, a normalization strategy is required. In Raman spectroscopy of bone tissue, different peaks can be considered to denote the mineral-to-matrix ratio, i.e. the mineralization degree of the ECM. In this study, we selected the phosphate stretching peak ($\nu_{1}{\mathrm{PO}}_{4}^{3-}$) normalized to the CH_2_ wagging integrated intensity, since it guarantees a good signal-to-noise ratio [39]. Moreover, we estimated the lipid-to-protein ratio by calculating the first spectral moment of the Raman CH_2_-CH_3_ band, using the relation

3.2 $$\bar{\upsilon }=\underset{i}{\sum }I_{i}\upsilon_{i}\;/\underset{i}{\sum }I_{i},$$

with $\upsilon_{i}$ ranging between 2800 and 3100 cm^−1^ [29,31].

#### 3.1.5. Scanning electron microscopy

SEM images were obtained using a Field Emission Scanning Electron Microscope LEO 1525 (ZEISS). Its InLens detector was used for the secondary electrons, while an AsB (Angle selective Backscattered) detector was used for the backscattered electrons. A layer of about 10 nm of chromium was deposited onto the region of interest within the sample, to prevent surface charging. Elemental mapping was performed using a Bruker Quantax EDX system equipped with a Peltier-cooled BRUKER XFlash 410-M silicon drift detector. EDX reveals the chemical composition of the sample through the analysis of X-ray energies emitted from the atoms upon interaction with the primary electrons.

## Data accessibility

Data generated through this work are made available at FigShare (doi:10.6084/m9.figshare.15135222) [56].
